# Genetic screen identifies a requirement for SMN in mRNA localisation within the *Drosophila* oocyte

**DOI:** 10.1186/s13104-018-3496-1

**Published:** 2018-06-13

**Authors:** Beppe Aquilina, Ruben J. Cauchi

**Affiliations:** 1 Department of Physiology and Biochemistry, Faculty of Medicine and Surgery, University of Malta, Msida, Malta; 2 Centre for Molecular Medicine and Biobanking, Biomedical Sciences Building, University of Malta, Msida, Malta

**Keywords:** Survival motor neuron, SMN, Genetic screen, Spinal muscular atrophy, mRNA localisation, *Gurken*, *Oskar*, *Encore*, *Syncrip*, *Hephaestus*

## Abstract

**Objective:**

Spinal muscular atrophy (SMA) results from insufficient levels of the survival motor neuron (SMN) protein. *Drosophila* is conducive to large-scale genetic-modifier screens which can reveal novel pathways underpinning the disease mechanism. We tested the ability of a large collection of genomic deletions to enhance SMN-dependent lethality. To test our design, we asked whether our study can identify loci containing genes identified in previous genetic screens. Our objective was to find a common link between genes flagged in independent screens, which would allow us to expose novel functions for SMN in vivo.

**Results:**

Out of 128 chromosome deficiency lines, 12 (9.4%) were found to consistently depress adult viability when crossed to *SMN* loss-of-function heterozygotes. In their majority, the enhancing deletions harboured genes that were previously identified as genetic modifiers, hence, validating the design of the screen. Importantly, gene overlap allowed us to flag genes with a role in post-transcriptional regulation of mRNAs that are crucial for determining the axes of the oocyte and future embryo. We find that SMN is also required for the correct localisation of *gurken* and *oskar* mRNAs in oocytes. These findings extend the role of SMN in oogenesis by identifying a key requirement for mRNA trafficking.

**Electronic supplementary material:**

The online version of this article (10.1186/s13104-018-3496-1) contains supplementary material, which is available to authorized users.

## Introduction

Spinal muscular atrophy (SMA) is a motor neuron disease caused by homozygous mutations in the *survival motor neuron 1* (*SMN1*) gene that are partially compensated by the paralogous *SMN2* gene. SMA patients have insufficient levels of the SMN protein, a situation triggering lower motor neuron degeneration and profound muscle weakness that restricts mobility and, in severe cases, results in respiratory failure and death [1]. SMN operates as part of a large multiprotein complex whose constituents also include Gemins 2–8 and Unrip [2]. The SMN complex is known to chaperone the assembly of ribonucleoproteins (RNPs) including small nuclear RNPs (snRNPs), which form the core components of the spliceosome [3], and messenger RNPs (mRNPs), which ensure transport as well as cytosolic localisation of mRNAs [4]. Whether either or both RNP assembly reactions are perturbed in SMA remains unclear. Animal models including the fruit fly *Drosophila melanogaster* are key for exploring the in vivo function of the SMN protein (reviewed in [5]). To this end, SMA-causing missense mutations (*SMN*^*73Ao*^) or deletion of the fly SMN gene orthologue leads to motor dysfunction in addition to defective neuromuscular junction (NMJ) morphology and transmission [6–8].

*Drosophila* is conducive to large-scale genetic-modifier screens which can potentially reveal novel pathways involved in the disease mechanism. The first *Drosophila* SMN genetic screen assessed whether a collection of transposon-induced mutations either enhanced or suppressed the lethality of *SMN*^*73Ao*^ heterozygotes and homozygotes, respectively. The identified modifier genes had no obvious role in RNP assembly with some including components of the bone morphogenetic protein (BMP) and fibroblast growth factor (FGF) signalling pathway [7, 9]. In a later study, the same lab performed a complementary screen this time using a hypomorphic *SMN* RNAi allele to increase sensitivity. A larger number of candidate genes that function in various pathways including RNA metabolism were successfully discovered [10]. Aiming at performing an independent SMN genetic screen, we tested the ability of a large collection of genomic deletions to reduce the viability of *SMN*^*73Ao*^ heterozygotes. To test our design, we asked whether our study can expose genomic regions containing genes identified in previous genetic screens. Finally, by exploring a common link between genes flagged in independent screens, we expose a function for SMN in post-transcriptional mRNA regulation in vivo.

## Main text

### Methods

#### Fly stocks

Flies were cultured on standard molasses/maizemeal and agar medium in plastic vials at an incubation temperature of 25 °C. The *SMN*^*73Ao*^ mutant has been characterised previously [6, 7, 11–14]. The chromosome 2 and 3 deficiency lines were obtained from the Bloomington *Drosophila* stock center at Indiana University, USA.

#### Genetic screen

Deficiency lines were crossed to the *SMN*^*73Ao*^ mutant line to determine whether haploinsufficiency of genomic regions have a negative influence on the adult viability of *SMN*^*73Ao*^ heterozygotes. Adult viability was calculated as the percentage number of adult flies eclosed divided by the expected number for the cross. For deficiencies that were found to depress adult viability, the cross was repeated for confirmation.

#### Bioinformatics

Genes mapped within the *SMN*^*73Ao*^-interacting chromosome deficiencies were listed using the ‘CytoSearch’ query tool on FlyBase [15] (http://flybase.org; FB2017_02 release). The ‘HitList’ tool was applied to the gene set to analyse the frequencies of values for gene ontology (GO) controlled vocabulary (CV) terms for biological process. GO enrichment analysis using the PANTHER classification system was performed using the enrichment analysis tool on the gene ontology consortium (GOC) website (http://geneontology.org).

#### Generation of mutant germline clones

The FLP-DFS (yeast flippase-dominant female sterile) technique (reviewed in [16]) was utilized to generate *SMN*^*73Ao*^ mutant germline clones. Virgin females having the *w*; *SMN*^*73Ao*^ *FRT2A/TM3*, *Ser* genotype were crossed to *y w hsFLP*; *ovo*^*D1*^ *FRT2A/TM3*, *Ser* males and recombination between the FRT (flippase recombinase target) sites in the resulting progeny was stimulated through heat-shock at 37 °C for 1 h at day 3, 4, and 5 after egg hatching. Egg chambers that survive beyond stage 4 in the ovaries of the female offspring (*y w hsFLP; SMN*^*73Ao*^ *FRT2A/ovo*^*D1*^ *FRT2A*) lack *ovo*^*D1*^ and are hence homozygous for *SMN*^*73Ao*^.

#### In situ hybridization

Ovaries were dissected in PBS (phosphate buffered saline) and later fixed in 4% paraformaldehyde in PBS at room temperature. Following treatment with proteinase K, ovaries were washed in PBS + 0.1% Tween20, re-fixed and washed again. They were later washed in a 1:1 solution formed of PBS + 0.1% Tween20: hybridization buffer (50% deionized formamide, 5× saline sodium citrate, 100 μg/ml *E. coli* tRNA, 50 μg/ml heparin, and 0.1% Tween20 in DEPC-water). Following pre-hybridisation for at least 1 h at 55 °C in hybridization buffer, DIG-labelled antisense *gurken* or *oskar* RNA probes were allowed to hybridise overnight in the same conditions. Three washing steps at 65 °C using (a) hybridisation buffer, (b) 1:1 PBS + 0.1% Tween20: hybridisation buffer, and (c) PBS + 0.1% Tween20 in that order, preceded incubation with sheep anti-DIG HRP-coupled antibody (1:2000; Roche Diagnostics Ltd.) for 2 h at room temperature. The hybridisation signal was amplified with Cy3-tyramide (PerkinElmer) and the ovaries were counterstained with Hoechst 33342 prior to mounting. Confocal images captured using the oil 40× magnification objective were processed using the ImageJ software (National Institutes of Health, Bethesda, MD, USA). Based on oocyte size and distinct mRNA localisation patterns, assessment was restricted to early stage 10 egg chambers.

### Results

**Fig. 1:**
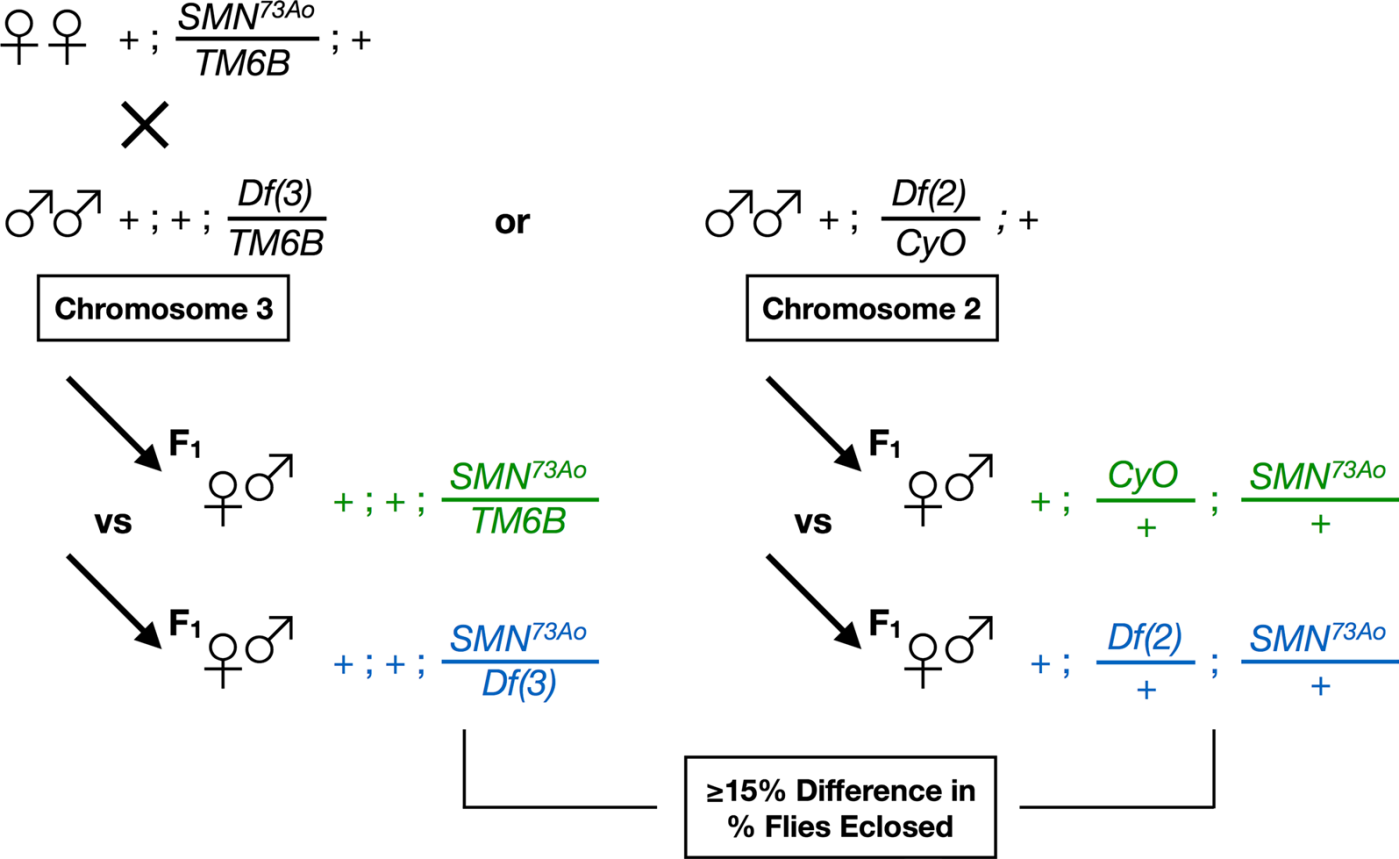
Schematic representation of the genetic screen. Individual second or third chromosome deficiencies were introduced in flies that are heterozygous for the *SMN*^*73Ao*^ loss-of-function allele. In the F1 generation, enhancing deletions were identified as those that reduced significantly the percentage number of flies eclosed when in *trans* with the *SMN*^*73A*o^ chromosome

To gain insights on pathways involved in SMA, we attempted at conducting a pilot genetic screen using part of the Bloomington Deficiency Kit. The Kit provides maximal coverage of the *Drosophila* genome with a minimal number of molecularly-defined deletions, hence facilitating genome-wide genetic screens [17]. Our screen involved a single stage designed to identify deletions that induced a pronounced decrease in adult viability when placed within an *SMN* loss-of-function heterozygous background. Previous studies suggested a strong association between the degree of adult viability and motor dysfunction phenotypes [7]. *SMN*^*73Ao*^*/TM6B* virgin females were mated to males carrying deletions spanning either arm of chromosome 3 (3R/3L) or the left arm of chromosome 2 (2L), hence, targeting approximately 50% of the *Drosophila* genome. In the F1 generation, flies of the appropriate genotype were identified to determine whether deletions placed in *trans* with the *SMN*^*73Ao*^ chromosome induced reduced viability compared to flies having the *SMN*^*73Ao*^ chromosome only (Fig. 1). The percentage number of flies eclosed was calculated and deletions were defined as ‘enhancers’ if they induced ≥ 15% difference, with the interaction strength being classified as mild (+, ≥ 15%), moderate (++, ≥ 25%), strong (+++, ≥ 35%) or intense (++++, ≥ 45%).

In total, 128 chromosome deficiency lines were evaluated and 12 (9.4%) were found to consistently depress the viability of *SMN* mutant heterozygotes (Table 1; Additional file 1: Table S1). The *Df(3L)81k19* deletion on the third chromosome produced the strongest enhancement, thereby leading to no adult viable flies. This was expected since one of the genes covered by the deficiency is the *SMN* gene, hence, *Df(3L)81k19* unsurprisingly failed to complement the loss-of-function *SMN*^*73Ao*^ mutation. Systematic evaluation of the candidate genes located within the genomic intervals flagged by the enhancing deletions is a laborious endeavour without a guarantee of success considering that more than one gene might be responsible for the enhanced phenotype. We therefore generated a ‘HitList’ formed of the genes uncovered by the enhancing deletions and probed the gene set for GO enrichment. Results were not statistically significant but some of the most frequent GO terms for biological process are pathways known to be disrupted in motor neuron disease including oxidation–reduction, neurogenesis, proteolysis, transcription, and translation [18] (Additional file 2: Table S2).

**Table 1:** Chromosome deficiency lines that depress the viability of *SMN*^*7Ao*^ heterozygotes

Chromosome	Deficiency	Deleted region	Deleted genes	% Flies eclosed mean ± SEM	Previously identified modifiers^a^	Interaction strength
2L	Df(2L)BSC37	22D1–22F2	74	84.1 ± 0.5	*tho2*	+
2L	Df(2L)ed1	24A2–24D4	66	76.7 ± 6.3	–	+
2L	Df(2L)BSC5	26B1–26D2	79	79.9 ± 5.4	*eIF4A*	+
2L	Df(2L)cact-255rv64	35F6–36D	181	83.1 ± 1.7	*VhaSFD*, *Tpr2*, *Sytα*	+
3L	Df(3L)HR119	63C6–63F7	75	81.5 ± 7.8	*enc*, *PIG*-*C*, *CG12016*, *PIG*-*B*, *CG32262*, *CG32263*, *CG32264*, *Rdh*, *CG42456*	+
3L	Df(3L)h-i22	66D10–66E2	29	66 ± 6.7	–	++
3L	Df(3L)vin5	68A2–69A1	239	71.1 ± 1.4	*Sod1*, *CG14130*, *Alg10*, *NaPi*-*III*	++
3L	Df(3L)81k19	73A3–74F4	175	0	*SMN*	++++
3R	Df(3R)WIN11	83E1–84A5	107	70.3 ± 8.7	*Dmtn*	++
3R	Df(3R)T-32	86D9–87C4	241	59.4 ± 7.2	*svp*, *GstD3*, *Cyp313a2*, *Jupiter*, *Csk*	+++
3R	Df(3R)BSC43	92F7–93B6	54	81.4 ± 3	*Syp*, *CG17272*	+
3R	Df(3R)B81	99D3–3Rt	280	48 ± 0.6	*heph*, *aralar1*, *CG9682*, *mRpL32*, *CG1750*	++++

^a^Genetic modifiers previously identified in the Chang et al. [7] and Sen et al. [10] studies

**Fig. 2:**
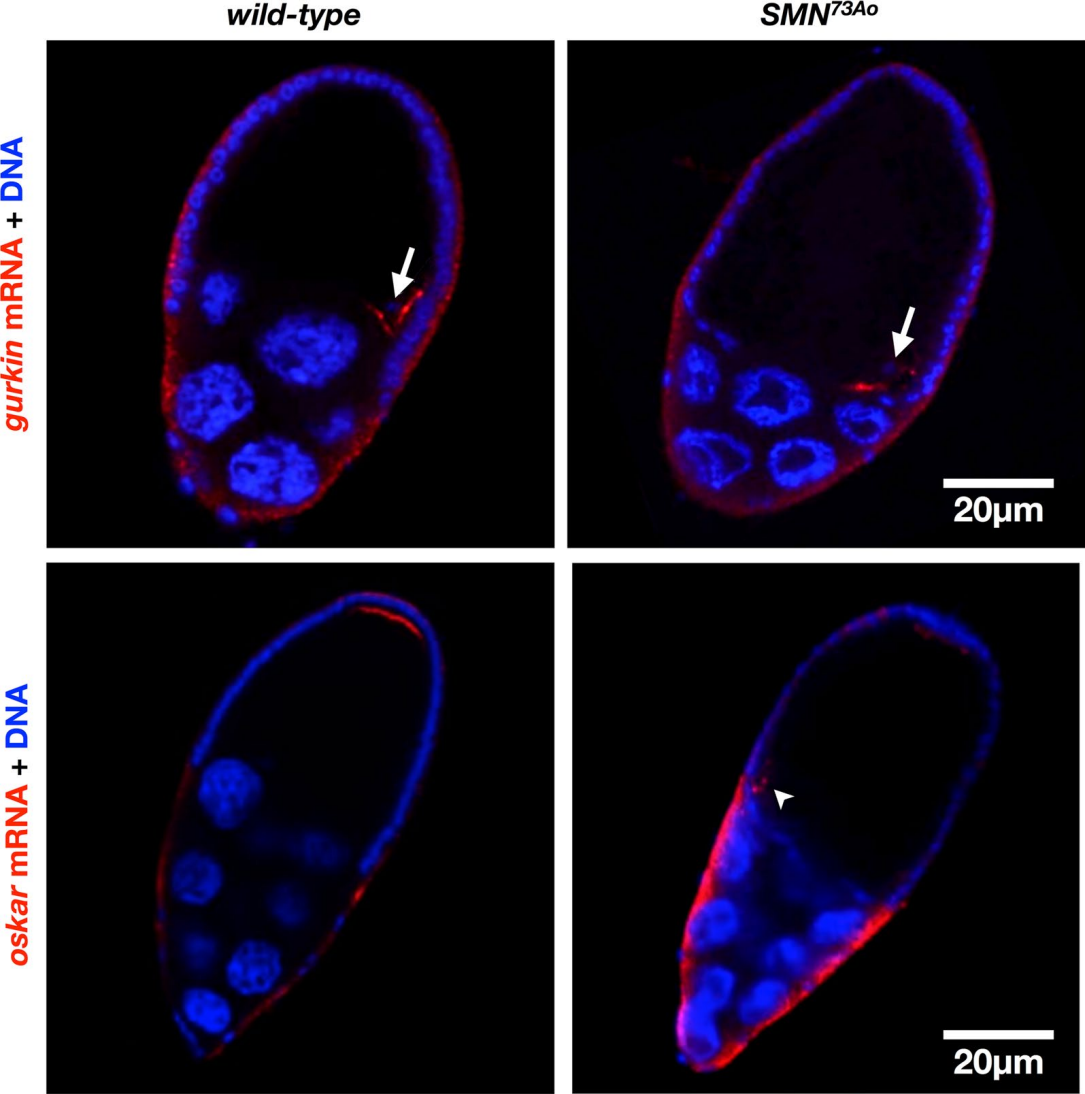
Aberrant mRNA localisation in *SMN*^*73Ao*^ mutant oocytes. Stage 10 egg chambers hybridised by either *gurken* or *oskar* antisense RNA probes and counterstained for DNA. Top is posterior whereas right is dorsal. In the top panel, arrows mark the oocyte nucleus; in the bottom panel, the arrow head marks residual transcript sometimes detected at the anterior corner

Interestingly, all the identified deletions with the exception of one (*Df[2L]ed1*), harboured genes that were previously found to modify SMN mutant phenotypes [7, 10]. In addition to validating the design of our screen, this finding can potentially flag genetic loci that overlap independently-conducted genetic screens. In this regard, we found a common thread running through 3 enhancing deletions. Each cover a previously identified genetic modifier that is known to have a role in post-transcriptional regulation of mRNAs that are crucial for determining the axes of the oocyte and future embryo [19]. The genes include *encore* (*enc*) covered by *Df(3L)HR119*, *Syncrip* (*Syp*) covered by *Df(3R)BSC43*, and *hephaestus* (*heph*) covered by *Df(3R)B81* (Table 1). Specifically, either gene was found to be required for the localisation of *gurken* and/or *oskar* mRNAs in oocytes [20–22]. Notably, considering the gene set uncovered by our genetic screen, oogenesis was also identified as one of the top-ranked most-frequent GO terms for biological process (Additional file 2: Table S2). The studies that have thus far explored a role for SMN in oogenesis have been few. Lee et al. [11] showed that defective nuclear organisation was the most prominent early defect in *SMN* mutant *Drosophila* eggs. We have previously observed similar phenotypes in egg chambers mutated for the SMN-associated DEAD-box helicase, Gemin3 [23, 24]. Considering our assessment of the genetic screen results, we asked whether SMN is also required for the correct localisation of *gurken* and *oskar* mRNAs. To this end, we find that in *SMN*^*73Ao*^ mutant oocytes, *gurken* mRNA was partially mis-localised, with transcript localisation skewed towards with the anterior or dorsal side (Fig. 2). This is in contrast to control oocytes in which *gurken* mRNA was always found tightly localised in a dorsal-anterior cap above the oocyte nucleus. Localisation of *oskar* mRNA was also defective. By the end of stage 8 of oogenesis, *oskar* mRNA accumulates in a crescent that is tightly localised to the posterior of the oocyte. In *SMN*^*73Ao*^ mutant oocytes, posterior oskar mRNA was only faintly detected (Fig. 2). Overall, these results extend the role of SMN in oogenesis by identifying a requirement for mRNA localisation.

### Discussion

In vivo studies have been supportive of a role for the SMN complex in snRNP assembly, hence, disturbances in this pathway and the consequential transcriptome abnormalities are thought to be the primary drivers of the progressive neuromuscular degeneration underpinning SMA (reviewed in [3]). In particular, we have previously shown that, in *Drosophila*, perturbation of snRNP assembly factors results in motor defects that mirror those described on loss of SMN or the Gemin constituents of the SMN complex [25–28]. Here, we exploited the genetic tractability of the fly system to identify genetic loci that influence SMN activity, thereby aiming at uncovering novel insights on SMN function in vivo. Thorough mining of the gene set uncovered by the *SMN* lethality-enhancing deletions allowed us to flag genes with a common function in RNA transport that were ‘hits’ in previous genetic screens. Making use of the extensively-studied *Drosophila* ovary, these findings led us to show that RNA transport is defective in SMN mutant oocytes. Although such phenotypes do not exclude a role for SMN in snRNP assembly, our results provide in vivo evidence implicating a function for SMN in RNA transport. This is corroborated by in vitro studies that are indicative of an involvement of SMN in mRNA trafficking within neurons (reviewed in [4, 29]).

Our study also extends the requirement of SMN during oogenesis. Hence, in addition to nuclear organisation and maintenance of the structural integrity of RNP bodies [11, 30], SMN is also crucial for the cytoplasmic localisation of mRNA transcripts that specify the future embryonic body axes. It is highly likely that the evident mislocalisation of *gurken* and *oskar* mRNAs contribute to the embryonic death observed for oocytes derived from an *SMN* mutant germline [6]. Our findings corroborate those by Grice and Liu [13] who showed that *SMN*^*73Ao*^ homozygous mutant neuroblasts failed to correctly localise the RNP component Miranda at the basal membrane. The exact function of SMN in mRNA trafficking remains unclear. Similar to its role in snRNP assembly, SMN might act as a molecular chaperone for the assembly of mRNP complexes [31]. The *Drosophila* ovary can however serve as a model system to further investigate the in vivo function of SMN in mRNA transport and localisation. Such studies can potentially provide insights on parallel activities occurring within the neuromuscular system and whose perturbation can lead to SMA.

## Limitations

Limitations arise from the lack of systematic evaluation of all the candidate genes covered by the enhancing deletions. In this regard, the contribution of previously identified genetic modifiers to the enhancing effect of the deletions is tentative.

## Additional files

**Additional file 1: Table S1.** Chromosome deficiency lines evaluated in the *SMN* enhancing screen.

**Additional file 2: Table S2.** Most frequent Gene Ontology (GO) terms for ‘biological process’ of genes covered by *SMN*^*73Ao*^ enhancing chromosome deficiencies. GO terms are ranked in descending order with #1 = most frequent and #14 = least frequent.

## Abbreviations

GO: gene ontology
SMA: spinal muscular atrophy
SMN: survival motor neuron
mRNP: messenger ribonucleoprotein
RNP: ribonucleoprotein
snRNP: small nuclear ribonucleoprotein
